# Measuring inhibitory control in children and adults: brain imaging and mental chronometry

**DOI:** 10.3389/fpsyg.2014.00616

**Published:** 2014-06-18

**Authors:** Olivier Houdé, Grégoire Borst

**Affiliations:** ^1^CNRS Unit 8140, Laboratory for the Psychology of Child Development and Education, Alliance for Higher Education and Research Sorbonne-Paris-Cité, Paris Descartes UniversityParis, France; ^2^Institut Universitaire de FranceParis, France

**Keywords:** inhibition, conceptual development, brain imaging, negative priming, number, categorization, logical reasoning

## Abstract

Jean Piaget underestimated the cognitive capabilities of infants, preschoolers, and elementary schoolchildren, and overestimated the capabilities of adolescents and even adults which are often biased by illogical intuitions and overlearned strategies (i.e., “fast thinking” in Daniel Kahneman’s words). The crucial question is now to understand why, despite rich precocious knowledge about physical and mathematical principles observed over the last three decades in infants and young children, older children, adolescents and even adults are nevertheless so often bad reasoners. We propose that inhibition of less sophisticated solutions (or heuristics) by the prefrontal cortex is a domain-general executive ability that supports children’s conceptual insights associated with more advanced Piagetian stages, such as number-conservation and class inclusion. Moreover, this executive ability remains critical throughout the whole life and even adults may sometimes need “prefrontal pedagogy” in order to learn inhibiting intuitive heuristics (or biases) in deductive reasoning tasks. Here we highlight some of the discoveries from our lab in the field of cognitive development relying on two methodologies used for measuring inhibitory control: brain imaging and mental chronometry (i.e., the negative priming paradigm). We also show that this new approach opens an avenue for re-examining persistent errors in standard classroom-learning tasks.

The scientific study of cognitive development in young children traces its roots back to Jean Piaget, a pioneer of this field in the 20th century (Piaget, 1954, 1983). Piaget described children as active learners who, through numerous interactions with their environments, construct a complex understanding of the physical world around them. From infancy to adolescence, children progress through four psychological stages: (1) the sensorimotor stage from birth to 2 years (when cognitive functioning is based primarily on biological reactions, motor skills and perceptions); (2) the preoperational stage from 2 to 7 years (when symbolic thought and language become prevalent, but reasoning is illogical by adult standards); (3) the concrete operations stage from 7 to 12 years (when logical reasoning abilities emerge but are limited to concrete objects and events); and (4) the formal operations stage at ~12 years (when thinking about abstract, hypothetical, and contrary-to-fact ideas becomes possible).

## FROM PIAGET’S THEORY TO INHIBITORY CONTROL MODEL

Piaget underestimated the cognitive capabilities of infants, preschoolers, and elementary schoolchildren, and he overestimated the capabilities of adolescents and adults, which are often biased by illogical intuitions and overlearned strategies (or heuristics) they fail to inhibit (Houdé, 2000, 2014; Kahneman, 2011). During the last three decades, detailed behavioral studies of children’s problem solving led to a reconceptualization of cognitive development, from discrete Piagetian stages to one that is analogous to overlapping waves (Siegler, 1996, 1999). The latter is consistent with a neo-Piagetian approach of cognitive development, in which more and less sophisticated solutions compete for expression in the human brain. In this approach, inhibition of less sophisticated solutions by the prefrontal cortex is a critical component of children’s conceptual insights associated with more advanced Piagetian stages (Houdé et al., 2000, 2011; Poirel et al., 2012; Borst et al., 2013a). According to this theoretical framework, the development of inhibitory control efficiency during childhood and adolescence contributes to the development of conceptual knowledge in various cognitive domains. This view is consistent with a number of studies showing that the dramatic development of the inhibitory control efficiency between 3- and 5-years old (e.g., Carlson, 2005) explains to some extent the growing ability of children to succeed in Theory of Mind (e.g., Benson et al., 2013), counterfactual reasoning (e.g., Beck et al., 2009) and strategic reasoning (e.g., Apperly and Carroll, 2009) tasks. In both of these literatures inhibition is viewed as a domain-general process allowing children and adults to resist habits or automatisms, temptations, distractions, or interference, and to adapt to conflicting situations (Diamond, 2013). Finally, in our view the gradual improvement of cognitive abilities in different domains is directly related to the improvement of inhibitory control efficiency. Note, however, that the development of inhibitory control efficiency is necessary but probably not sufficient to produce conceptual development during childhood and adolescence.

At any point in time, children and adults potentially have available to them heuristics (i.e., intuitions) and logicomathematical algorithms, or as Kahneman (2011) described, multiple levels of “thinking fast and slow.” Heuristics are rapid, often global or holistic, useful strategies in many situations, *but sometimes they are misleading*, whereas algorithms are slow, demanding and analytical strategies that *necessarily lead to a correct* (i.e., *logical*) *solution in every situation*. In general, children and adults prefer using fast heuristics spontaneously, but that choice does not indicate that they are illogical *per se* (Houdé, 2000) or that they are “happy fools” (De Neys et al., 2013, 2014). Psychologists had to be careful to avoid false negatives (Gelman, 1997), which is a strong tendency to say that those children or adults who fail a task are incompetent in the target domain of knowledge. A “presumption of rationality” is sometimes the best assessment.

Contrary to Piaget’s theory, infants learn more about the outside world through information that is captured by their perceptual systems than through motor skills development (Mandler, 1988; Baillargeon, 1995; Spelke, 2000). Infant cognition studies evaluated both the capacity to interpret sensory data and the faculty for understanding and reasoning about complex events. In the last 10 years, theoretical ideas and empirical research in the field have demonstrated that very young children’s learning and thinking mechanisms do remarkably resemble the basic inductive processes of science, i.e., probabilistic models and Bayesian learning methods (Gopnik, 2012). Infants can implicitly reason statistically (Téglas et al., 2011). From this point of view, the very young child is already seen as a “scientist in the crib” (Gopnik et al., 1999).

## THE NUMBER EXAMPLE

A heated debate topic in psychology is how children come to understand numbers (Dehaene, 1997). Piaget’s answer was that number is constructed in children through the logicomathematical synthesis of classification and seriation operations (Piaget, 1952). Number borrows its inclusion structure from classes (1 is included in 2, 2 in 3, etc.); because it disregards qualities by transforming objects into units, it brings a serial order into play, the sole means of distinguishing one unit from the next: 1 then 1, then 1, etc. The serial ordering of units is combined with the inclusion of the sets that result from their union (1 is included in 1 + 1, 1 + 1 is included in 1 + 1 + 1, etc.) to constitute number. The task Piaget used was conservation of number. When children are shown two rows of objects that contain an equal number of objects but that differ in length (because the objects in one of the rows have been spread apart), young children think the longer row has more objects. Piaget’s interpretation was that preschool children are still fundamentally intuitive (their reasoning is illogical by adult standards), or as he called them, “preoperational” (Stage 2), and hence limited to a perceptual way of processing information (based on length or, in certain cases, on density). At the age of 6 or 7 years old, children understand the equivalency of quantities, regardless of apparent transformations. At this point, they are called “operational” or “conserving” (Stage 3), the criterion for mastery of number. Piaget also worked on determining whether the conservation of number develops simultaneously with inclusion (classification) and order relations (seriation).

After this founding work on the genesis of number, research in this domain proliferated, and criticisms of Piaget’s theory were far from scarce. First, the synchronous development of classification, seriation, and conservation was not validated in experimental verifications. Second, it became clear that Piaget’s view of the logico-structural aspect of number is overly polarized and overshadows the more functional aspects of numerical development, such as counting.

A radical change in perspective began with Gelman and Meck (1983), Gelman et al. (1986), who not only turned the attention toward counting but also postulated the early existence of five fundamental principles of counting: stable order (order of the number words), strict one-to-one correspondence (between the number words and the items counted), cardinality (the number word corresponding to the last item counted is equal to the total number of items), abstraction (any kind of item can be counted), and order irrelevance (items can be counted in any order). Gelman demonstrated the presence of these principles in young children by having them say whether they thought a doll was counting correctly or incorrectly. Knowledge or lack of knowledge of a given principle was deduced from whether the child detected the corresponding type of counting error (unstable order, violation of the one-to-one correspondence, cardinal number referred to by an ordinal word number, etc.). The results indicated that 3-year-old children have already acquired the basic principles of counting. This led Gelman to distinguish three components in the ability to count: a conceptual component (“knowing why” or understanding the five principles), a procedural component (“knowing how” or understanding the structure and order of counting), and a utilization component (“knowing when” or understanding the relevance of using the first two components in a given context). Defending the principles-before-skills hypothesis, Gelman suggested that the numerical difficulties of preschool children lie essentially in the procedural and utilization components. Another of Gelman’s original contributions was her use of the so-called “magic task” to demonstrate that 3- to 4-year-old children are surprised by transformations that affect the cardinal number of a set (adding and subtracting items) but not by transformations that do not (spreading and grouping) (Gelman, 1972). She concluded that despite their failure in Piaget’s conservation of number task, the children at this age are already capable of seeing through irrelevant transformations and treating the number of items as invariable (for a seminal study on this point, see Mehler and Bever, 1967). This new conclusion was corroborated by the discovery of the perception of numerical invariance in neonates (Antell and Keating, 1983) and in 5- and 8-month-old infants (Loosbroek and Smitsman, 1990; Lipton and Spelke, 2003).

The most striking example of infants as “mathematicians” is found in the famous work by Wynn (1992). Wynn recorded the looking time of 4- and 5-month-old infants in the “impossible-event” procedure (or violation-of-expectation procedure) and demonstrated that infants were surprised by (looked longer at) impossible numerical events (e.g., 1 + 1 = 1 and 1 + 1 = 3, or 2 - 1 = 2) but were not surprised at the corresponding possible events (1 + 1 = 2 and 2 - 1 = 1; the events were staged with Mickey Mouse figures). She concluded that infants are endowed with a mechanism that calculates the exact outcome of simple arithmetic operations. She claimed that infants at this age are already able to encode ordinal information and possess genuine numerical concepts that cannot be reduced to holistic percepts derived from a pattern recognition process (for a brain imaging confirmation of Wynn’s results, see Berger et al., 2006). Like Gelman’s stance, Wynn’s position is strong and seems to run counter to what we know about the numerical difficulties of preschool children. Wynn’s empirical results are robust and consistent (Wynn, 2000), but they have sparked theoretical debates (Simon, 1997, 1998). The task of the following research was to devise a developmental model of logicomathematical operations (conservation, counting, and elementary arithmetic) that accounts for both early abilities (Gelman, Wynn, etc.) and late inabilities (Piaget), without denying the reality of the former but raising the question of the factors that explain the latter.

## HEURISTICS, ALGORITHMS, COGNITIVE CONTROL, AND INHIBITION OF MISLEADING STRATEGIES

How might we explain the famous number-conservation error observed in children until the age of seven by Piaget and, after him, by all developmental psychologists around the world? It is an intriguing question because we know today that very young children are already capable of treating the number of items as invariable through irrelevant transformations and that they possess other protonumerical skills. One of the main current explanations is that children learn heuristics, which are often useful in a large set of situations, but fail to inhibit them when, contrary to general practice, they are misleading (Houdé, 2000, 2014). In the case of Piaget’s number-conservation algorithm, the overlearned competing heuristic is “length-equals-number” (Houdé and Guichart, 2001). This new theoretical approach is in line with Diamond’s explanation of the A-not-B error in infants (see Diamond, 1991) and assumes that cognitive development relies not only on the acquisition of knowledge of incremental complexity (Piaget, 1983) but also on the ability to inhibit previously acquired knowledge (Bjorklund and Harnishfeger, 1990; Diamond, 1991, 1998; Dempster and Brainerd, 1995; Harnishfeger, 1995; Houdé, 2000). Increasing evidence shows that the ability to inhibit previous knowledge is critical for developmental milestones, such as those defined by Piaget’s theory (Borst et al., 2013a; Houdé, 2014). Inhibitory control of misleading strategies, an executive function performed by the prefrontal cortex, has been claimed necessary for acquisition and use of motor or cognitive algorithms in the fields of object permanence in infants (Diamond and Goldman-Rakic, 1989; Diamond, 1991, 1998; Bell and Fox, 1992), number-conservation and class inclusion in preschool and schoolchildren (Houdé and Guichart, 2001; Perret et al., 2003; Borst et al., 2012, 2013b), and logical reasoning in adolescents and adults (Houdé et al., 2000; Houdé and Tzourio-Mazoyer, 2003; Houdé, 2007).

One of the challenges of today’s developmental research, in all domains of cognition ranging from motor programming to high-order logical reasoning, is to account not only for a general and incremental process of coordination-activation capacities of structural units, schemes or skills through ages and stages (Piaget, 1983, and all the 1980s neo-Piagetians: see the review book by Demetriou, 1988) but also for a general process of selection-inhibition of competing strategies, i.e., heuristics (or intuitions) and logicomathematical algorithms, occurring with different weights at any point in time, depending on the context, in a non-linear dynamical system of growth (Siegler, 1996, 1999; Houdé, 2000, 2014). Such cognitive model introduces less regular developmental curves containing perturbations, bursts, and collapses. O’Reilly (1998) described six principles for biologically based computational models of cognition, one of which is inhibitory competition (see also Johnson, 2010). Resolving this “inhibition issue” is an important task for both developmental psychology and cognitive neuroscience. The most compelling magnetic resonance imaging (MRI) reports of structural changes with brain development during childhood and adolescence showed a sequence in which the higher-order association area, such as the prefrontal cortex sustaining inhibitory control, matures last (Casey et al., 2005). The sequence in which the cortex matures parallels the cognitive milestones in human development. First, the regions subserving primary functions, such as motor and sensory systems, mature the earliest; the temporal and parietal association cortices associated with basic language skills and spatial attention mature next; and the last to mature are the prefrontal cortex and its inhibitory control ability.

## BRAIN IMAGING: INHIBITORY CONTROL AND PREFRONTAL CORTEX

Using fMRI (functional magnetic resonance imaging), from this theoretical perspective, we re-examined what occurs in the developing brain when school children are tested for their performance in Piaget’s number-conservation task. Remember that when children are shown two rows of objects that contain an equal number of objects but that differs in length (because the objects in one of the rows had been spread apart), young children think that the longer one has more objects. Piaget’s interpretation was that preschool children are still fundamentally intuitive (their reasoning being illogical by adult standards), or as he called them, “preoperational” (Stage 2), and hence limited to a perceptual way of processing information (here, based on length or, in certain cases, on density). When they are ~6 or 7 years old, children understand the equivalency of quantities, regardless of apparent transformations. At this point, they are called “operational” or “conserving” (Stage 3), the criterion for logicomathematical mastery of number. Our new hypothesis was that their main cognitive difficulty (beyond logicomathematical cognition *per se*) was to efficiently inhibit through their prefrontal cortex the overlearned “length-equals-number” strategy, a heuristic that is often used both by children and adults in many school and everyday situations.

In a first fMRI study, we found that the cognitive change allowing children to access conservation (i.e., the shift from Stage 2 to Stage 3 in Piaget’s theory) was related to the neural contribution of a bilateral parietofrontal network involved in numerical and executive functions (Houdé et al., 2011). These imaging results highlighted how the behavioral and cognitive stages that Piaget formulated during the 20th century manifest in the brain with age. In a second fMRI study (Poirel et al., 2012), we demonstrated that the prefrontal activation (i.e., the blood-oxygen-level-dependent signal) observed when schoolchildren succeeded at the Piaget’s number-conservation task was correlated to their behavioral performance on a Stroop-like measure of inhibitory function development (Wright et al., 2003). These new results in schoolchildren fit well with previous brain imaging data from our laboratory showing a key role of prefrontal inhibitory control training when adolescents or adults (belonging to Stage 4 in Piaget’s theory) spontaneously fail to block their perceptual intuitions (or bias, heuristics) to activate logicomathematical algorithms (i.e., deductive rules) in reasoning tasks (Houdé et al., 2000; Houdé, 2007).

If we have “two minds in one brain” as stated by Evans (2003) or, in other words, two ways of thinking and reasoning, i.e., “fast and slow” (Kahneman, 2011), currently called “System 1” (intuitive system) and “System 2” (analytic system), then the crucial challenge is to learn to inhibit the misleading heuristics from System 1 when the more analytic and effortful System 2 (logicomathematical algorithms) is the way to solve the problem (Houdé, 2000, 2014; Borst et al., 2013a). Within this post-Piagetian theoretical approach, we can now understand why, despite rich precocious knowledge about physical and mathematical principles observed in infants and young children, older children, adolescents, and adults so often have poor reasoning. The cost of blocking our intuitions is high and depends on the late maturation of the prefrontal cortex. Moreover, this executive ability remains delicate throughout our lifetime, and adults may sometimes need “prefrontal pedagogy” to learn inhibiting intuitive heuristics (or biases) in reasoning tasks (Houdé, 2007).

An innovative research question now is to better understand the cognitive roots of such powerful heuristics (intuitions and bias from System 1) that children and adults have so much difficulty inhibiting in some cases. New heuristics may appear and be overlearned at any time in the course of development (Houdé, 2000, 2014) because our brain is an irrepressible detector of regularities from its perceptual and cultural environment. For example, preschool children (more than infants) are often exposed, in “math books” in the classroom or in everyday scenes, to patterns of objects in which number and length covary (e.g., the 1-to-10 Arabic numbering series is frequently illustrated by increasing lines of drawn animals or fruits: one giraffe, two hippopotamus, three crocodiles, and so on), hence the overlearned and misleading “length-equals-number” heuristic, which is overactivated in Piaget’s conservation of number task. A new avenue of research would be to assess the role of early sensitivity to statistical patterns (i.e., probability of hypotheses) and Bayesian inference (Gopnik, 2012) in the psychological construction of perceptual, motor, and cognitive heuristics. Moreover, the power of Bayesian learning might require, in some conflict situations, a strong antagonist process of inhibition for blocking heuristics when they are misleading.

## MENTAL CHRONOMETRY: INHIBITORY CONTROL AND NEGATIVE PRIMING EFFECT

In this section, we will review mental chronometry studies that used negative priming to demonstrate the role of inhibitory control in logicomathematical tasks. The logic of the negative priming approach is as follows: if information (or a perceptual or cognitive heuristic) was previously ignored (or inhibited), then the subsequent processing of that information (or the subsequent activation of that heuristic strategy) will be disrupted as revealed by slower or less accurate responses (see, e.g., Tipper, 1985, 2001; Neill et al., 1995). In the classical negative priming paradigm, participants performed pairs of stimuli. The first stimulus of the pair is the prime; the second one is the probe. Classically, participants’ performance is measured on the second stimulus (i.e., probe). Critically, performance are compared between test-probes in which the target is a distractor inhibited on the first stimulus (i.e., prime) and control-probes in which the target bears no relation with a distractor inhibited on the prime. The logic of the negative approach is similar for strategies: if to reason logically one need to inhibit an overlearned strategy (or heuristic) to activate a logical algorithm, then a negative priming effect should be observed when participants perform prime-probe sequences in which the heuristic that needs to be activated on the probe was inhibited on the prime. Bluntly put, if people block the heuristic response on one trial, they will pay a price if they need to rely on it on the subsequent trial.

Following this logic, Houdé and Guichart (2001) devised the first negative priming paradigm to demonstrate that inhibitory control was required when children correctly solved a classic logicomathematical task – Piaget’s number-conservation task (Piaget, 1952). The authors asked children to perform two types of prime-probe trials. In test trials, two rows of different length but with the same number of objects (i.e., a classical number-conservation item) were presented as the prime. In order to correctly state that the two rows contained the same number of objects, children had to inhibit the length-equals-number heuristic. On the probe, an item in which length and number co-varied – i.e., the longer row contained more objects – was displayed. Critically, the length-equals-number strategy that was inhibited on the prime became the appropriate strategy to activate on the probe. In control trials, the strategy to be used on the prime was unrelated to the strategy to activate on the probe. Objects were displayed in such a way that counting each object was the only appropriate strategy (i.e., the objects on one of the rows were displayed vertically on the screen which ruled out using the length-equals-number strategy). As on the test trials, an item in which length and number co-varied was displayed on the probe. Comparison of the probe response times between test and control trials revealed a clear negative priming effect: children were slower to use the length-equals-number strategy after they performed a typical Piaget-like number-conservation item in which the length-equals-number heuristic needs to be inhibited to overcome the interference between the length of the rows and the number of objects. This result suggests that children’s ability to reason correctly on number-conservation tasks is directly related to their ability to inhibit a misleading strategy.

Note that as opposed to Piaget’s seminal number-conservation task, the transformation (i.e., the lengthening of one of the rows) is not presented to the children in Houdé and Guichart (2001)’ study (due to the inherent structure of such sequential paradigm). Thus, one could claim that Piaget’s seminal number-conservation task and the Piaget-like number-conservation task designed by Houdé and Guichart test very different numerical knowledge of the children and (b) that success at Piaget’s seminal number-conservation task might have nothing to do with the inhibition of the length-equals-number strategy. However, recent fMRI and high-density EEG studies (Houdé et al., 2011; Poirel et al., 2012; Borst et al., 2013c) revealed that children and adults must inhibit the length-equals-strategy to succeed at Piaget’s seminal number-conservation task in agreement with the results reported on the Piaget-like number-conservation task designed by Houdé and Guichart (2001).

In a follow-up electrophysiological study using a similar negative priming adaptation of the number-conservation task with young adults, Daurignac et al. (2006) reported enhanced amplitude of the N200 wave (with a large distribution over the scalp) when the length-equals-number strategy inhibited on the prime became the appropriate strategy to activate on the probe. Given that the N200 is assumed to reflect inhibitory control, electrophysiological data garnered in this study suggest that adults as children need to inhibit the length-equals-number heuristic to reason correctly here (see Borst et al., 2013c for an incremental demonstration using high-density ERP).

Negative priming has also been reported in another famous logicomathematical Piagetian task, the class-inclusion task (Inhelder and Piaget, 1964). In this task, ten daisies (i.e., the subordinate class A) and two roses (i.e., the subordinate class A’) are presented to the child and he(she) is asked whether there are more daisies than flowers (i.e., the superordinate class B = A+A’). Before the age of seven, children erroneously think that there are more daisies than flowers because they fail to perform the appropriate comparison between the superordinate class (flowers) and the subordinate class (daisies). To succeed at this task, children need to inhibit the direct (heuristic) perceptual comparison of the visuospatial extensions (the number of displayed elements) of the two subclasses (A and A’) in order to activate the appropriate logical (or conceptual) comparison of the superordinate class (B) to its subordinate class (A) – the class-inclusion algorithm. In the negative priming adaptation of the class-inclusion task, adults and 10-year-old children performed test and control trials with three types of items: class-inclusion items, subclasses-comparison items, and control items (Borst et al., 2013b). Stimuli consisted of two rows of various geometric shapes of different colors separated by a horizontal line (e.g., eight green squares and four blue squares). Class-inclusion items (e.g., “More green squares than squares”: yes or no?) required to compare the superordinate class (e.g., squares) to one of its subordinate classes (e.g., green squares). Subclasses-comparison items required to compare the number of elements in the two subclasses (e.g., “More green squares than blue squares”). On control items participants were required to judge whether all objects had the same given property (e.g., “Squares have the same color”). In the test trials, participants performed a typical class-inclusion item on the prime (in which inhibition of the comparison of the subordinate classes’ extensions was needed) and then a subclasses-comparison item on the probe (in which the direct comparison of the two subclasses’ extensions became the appropriate strategy, e.g., comparing the number of blue and green squares). In the control trials, participants performed a control item on the prime followed by a subclasses-comparison item on the probe. Critically, the strategy to be used on the prime was not related to the strategy to be used on the probe. Negative priming was reported for both children and adults: children and adults were slower to determine that there were more objects in one subordinate class than in the other after they successfully determine that there were more elements in the superordinate class than in one of the two subordinate classes. In addition, negative priming decreased with age. The results reported in this study extend the related findings of Perret et al. (2003) in school-aged children by showing (a) that young adults still need to inhibit the misleading perceptual strategy – i.e., the direct comparison of the subordinate classes – to reason about class inclusion and (b) that the efficiency of the inhibitory control needed in this specific task increases between fourth graders and young adults.

Another study from our lab has used a negative priming paradigm to demonstrate that inhibition is required in syllogistic reasoning (Moutier et al., 2006; Borst et al., 2013a). As in other negative priming studies, children performed test and control trials. Each trial consisted of two syllogisms with many words in common. In test trials, on the prime the validity of the syllogism was in contradiction with children’s knowledge of the world (e.g., *All elephants are light*). Therefore, children had to inhibit their belief heuristic (e.g., elephants are heavy) to correctly judge the logical validity of the conclusion. On the probe, a syllogism was presented in which children’s belief was congruent with the logical validity of the conclusion (e.g., *All elephants are light*, when the conclusion was not valid). Critically, the belief that was inhibited on the prime was congruent with the validity of the syllogism on the probe. On control trials, children solved neutral syllogisms in which the conclusion was neither unacceptable nor acceptable regarding the children’s beliefs (e.g., *No students in the blue school are interested in sports*) followed, on the probe, by a syllogism in which the belief was congruent with the logical validity of the conclusion. As expected if inhibitory control is needed for syllogistic reasoning a negative priming effect was reported on the number of errors made by the participants: children committed more errors on the congruent syllogisms (probe items) when performed after syllogisms (prime items) in which beliefs and the validity of the conclusion interfered. Thus, as with the other logicomathematical tasks that we reviewed, syllogistic reasoning seems directly related to the ability to inhibit irrelevant strategies (or beliefs) in order to activate a logical algorithm.

Further studies are needed to investigate whether inhibitory control development during childhood and adolescence contributes to conceptual development in other cognitive domains than the ones we investigated (i.e., number, categorization, and reasoning).

## FROM THE LAB TO THE CLASSROOM

Finally, beyond classical laboratory experimental situations, it seems that some systematic difficulties children have in resolving problems in the classroom are also related to their difficulty to inhibit what they previously learned. For example, we investigated whether simple arithmetic word problems such as “Bill has 20 marbles. He has five more marbles than John. How many marbles does John have?” could remain challenging for children because they fail to inhibit the “add if more, subtract if less” misleading heuristic. Indeed, errors in this type of problems are characterized by adding the numbers instead of subtracting them or vice versa. Using a negative priming paradigm, we demonstrated that children and even adults must inhibit the “add if more, subtract if less” misleading strategy to solve simple arithmetic word problems in which the relational term (“more” or “less”) is incongruent with the arithmetic operation to perform (Lubin et al., 2013). Thus, the increased efficiency to solve this type of problems from childhood to adulthood may be directly related to the gradual development of inhibitory control efficiency.

This new approach of cognitive development opens an avenue for designing pedagogical interventions (in line with Zelazo, 2006; Diamond et al., 2007; Houdé, 2007; Chevalier and Blaye, 2008; Diamond and Lee, 2011; Moriguchi, 2012) based on training the inhibition of heuristics (or reasoning biases). Previous studies have demonstrated that this type of pedagogical interventions not only improve logical reasoning to a greater extent than ones based solely on verbal logic *per se* (e.g., Houdé et al., 2000; Moutier and Houdé, 2003; Houdé, 2007; Cassotti and Moutier, 2010) but also help children in the classroom overcome systematic difficulties to a greater extent than traditional curricula (Lubin et al., 2012). Further studies much as the ones conducted on the effect of training working memory, another domain-general executive function (see e.g., Olesen et al., 2004), are needed to determine more precisely the effect of inhibitory control training on the development of the prefrontal cortex.

## Conflict of Interest Statement

The authors declare that the research was conducted in the absence of any commercial or financial relationships that could be construed as a potential conflict of interest.
